# Prediction of Inflammatory Breast Cancer Survival Outcomes Using Computed Tomography-Based Texture Analysis

**DOI:** 10.3389/fbioe.2021.695305

**Published:** 2021-07-20

**Authors:** Sung Eun Song, Bo Kyoung Seo, Kyu Ran Cho, Ok Hee Woo, Balaji Ganeshan, Eun Sil Kim, Jaehyung Cha

**Affiliations:** ^1^Department of Radiology, Korea University Anam Hospital, Korea University College of Medicine, Seoul, South Korea; ^2^Department of Radiology, Korea University Ansan Hospital, Korea University College of Medicine, Ansan, South Korea; ^3^Department of Radiology, Korea University Guro Hospital, Korea University College of Medicine, Seoul, South Korea; ^4^Institute of Nuclear Medicine, University College London Hospitals NHS Trust, London, United Kingdom; ^5^Medical Science Research Center, Korea University Ansan Hospital, Korea University College of Medicine, Ansan, South Korea

**Keywords:** overall survival, breast neopalsms, computed tomgraphy, texture, histogram

## Abstract

**Background:** Although inflammatory breast cancer (IBC) has poor overall survival (OS), there is little information about using imaging features for predicting the prognosis. Computed tomography (CT)-based texture analysis, a non-invasive technique to quantify tumor heterogeneity, could be a potentially useful imaging biomarker. The aim of the article was to investigate the usefulness of chest CT-based texture analysis to predict OS in IBC patients.

**Methods:** Of the 3,130 patients with primary breast cancers between 2006 and 2016, 104 patients (3.3%) with IBC were identified. Among them, 98 patients who underwent pre-treatment contrast-enhanced chest CT scans, got treatment in our institution, and had a follow-up period of more than 2 years were finally included for CT-based texture analysis. Texture analysis was performed on CT images of 98 patients, using commercially available software by two breast radiologists. Histogram-based textural features, such as quantification of variation in CT attenuation (mean, standard deviation, mean of positive pixels [MPP], entropy, skewness, and kurtosis), were recorded. To dichotomize textural features for survival analysis, receiver operating characteristic curve analysis was used to determine cutoff points. Clinicopathologic variables, such as age, node stage, metastasis stage at the time of diagnosis, hormonal receptor positivity, human epidermal growth factor receptor 2 positivity, and molecular subtype, were assessed. A Cox proportional hazards model was used to determine the association of textural features and clinicopathologic variables with OS.

**Results:** During a mean follow-up period of 47.9 months, 41 of 98 patients (41.8%) died, with a median OS of 20.0 months. The textural features of lower mean attenuation, standard deviation, MPP, and entropy on CT images were significantly associated with worse OS, as was the M1 stage among clinicopathologic variables (all *P-*values < 0.05). In multivariate analysis, lower mean attenuation (hazard ratio [HR], 3.26; *P* = 0.003), lower MPP (HR, 3.03; *P* = 0.002), and lower entropy (HR, 2.70; *P* = 0.009) on chest CT images were significant factors independent from the M1 stage for predicting worse OS.

**Conclusions:** Lower mean attenuation, MPP, and entropy on chest CT images predicted worse OS in patients with IBC, suggesting that CT-based texture analysis provides additional predictors for OS.

## Introduction

Inflammatory breast cancer (IBC) is an aggressive malignancy and accounts for 1–6% of all breast cancers (Anderson et al., 2005). Despite the lack of molecular markers that distinguish inflammatory and non-inflammatory breast cancer at the molecular level, the two clinical entities are clearly distinct in terms of symptoms, natural history, and survival (Fouad et al., 2017). It is characterized by diffuse edema and erythema, involving one-third or more of the skin of the breast that results from tumor infiltration of the dermal lymphatics. IBC is classified as “cT4d” by the American Joint Committee on Cancer (AJCC) (Giuliano et al., 2018). Thus, patients with non-metastatic IBC are considered stage IIIB, regardless of tumor size or lymph node spread, and the patients with metastatic IBC are considered stage IV. Fouad et al. (2015) reported that the patients with IBC who present with distant metastasis at diagnosis (stage IV) have significantly worse overall survival compared with those who have stage IV non-inflammatory breast cancer. With the introduction of trimodal treatment, including neoadjuvant chemotherapy, mastectomy, and radiation therapy, the prognosis of IBC has improved with a 2-year survival rate of 62% between 1990 and 1995 to 76% between 2006 and 2010 (Ueno et al., 1997; Low et al., 2004; Dawood et al., 2014). However, even after trimodal treatment, IBC still has a very poor outcome with a 5-year overall survival (OS) rate of 25–50% (Hance et al., 2005; Cristofanilli et al., 2007). Therefore, it is important to identify early prognostic factors that predict poor survival outcomes and to determine aggressive treatment in a carefully selected group of IBC patients.

Several studies demonstrated that diffuse erythema, lymph node involvement, chest-wall adherence, hormone receptor-negative status, age >50 years, and incomplete tumor regression after chemotherapy are associated with worse survival outcomes in IBC (Chevallier et al., 1987; Fields et al., 1989; Palangie et al., 1994; Pierga et al., 2017). Therefore, breast imaging is crucial in determining the extent of tissue involvement and predicting the prognosis of IBC. However, few studies have used imaging features to predict the prognosis in IBC patients. To assess AJCC cancer staging and treatment effectiveness according to the response evaluation criteria in solid tumors (RECIST) (Eisenhauer et al., 2009), morphological methods, such as tumor size measurements on imaging, are essential. However, IBC regards as a non-measurable tumor at the RECIST guideline, and tumor size measurement is inappropriate for evaluation of the treatment response. Therefore, there is an unmet need for treatment monitoring and risk stratification of patients with IBC.

Texture analysis, which is a non-invasive technique that quantifies the spatial pattern of pixel intensities on imaging, is an objective measure of tumor heterogeneity (Ganeshan et al., 2013). Tumor heterogeneity has been recognized as a key feature of therapeutic resistance that reflects intra-tumor variation in cell density, necrosis, or angiogenesis (Nelson et al., 2004). Computed tomography (CT)-based texture analysis has been shown to provide predictive information about survival or recurrence or response to chemotherapy in patients with lung, liver, head and neck, and colorectal cancer (Ganeshan et al., 2012; Ng et al., 2013; Zhang et al., 2013; Chee et al., 2017; Mule et al., 2018). However, there is very little published literature on CT-based texture analysis in breast cancer despite several studies have demonstrated that textural features from magnetic resonance imaging (MRI) are associated with survival outcomes (Pickles et al., 2016; Kim et al., 2017) and response to neoadjuvant chemotherapy (Chamming's et al., 2018). Because IBC is considered at least stage IIIB at diagnosis, systemic staging studies, such as chest CT or positron emission tomography, combined with CT scan, are done to assess whether the cancer has spread to lymph nodes or lungs. It was reported that 95% of patients with stages III and IV breast cancer underwent chest CT scan for staging (James et al., 2019). That study shows that chest CT is one of the investigations of choice for staging and metastatic work-up of new breast cancers, and suggests routine performance of chest CT in advanced breast cancers (stages III and IV) because metastases are higher compared with early breast cancers (stages I and II). If simpler measures (e.g., histogram analysis) of CT-derived prognostic biomarkers could be readily incorporated in routine clinical practice, they could be potentially useful non-invasive imaging biomarkers, which could help in making optimal treatment decisions and improve overall clinical outcomes.

The purpose of this study was to investigate the usefulness of CT-based texture analysis and clinicopathologic features to predict OS in patients with IBC.

## Methods

### Patients

**Figure 1:**
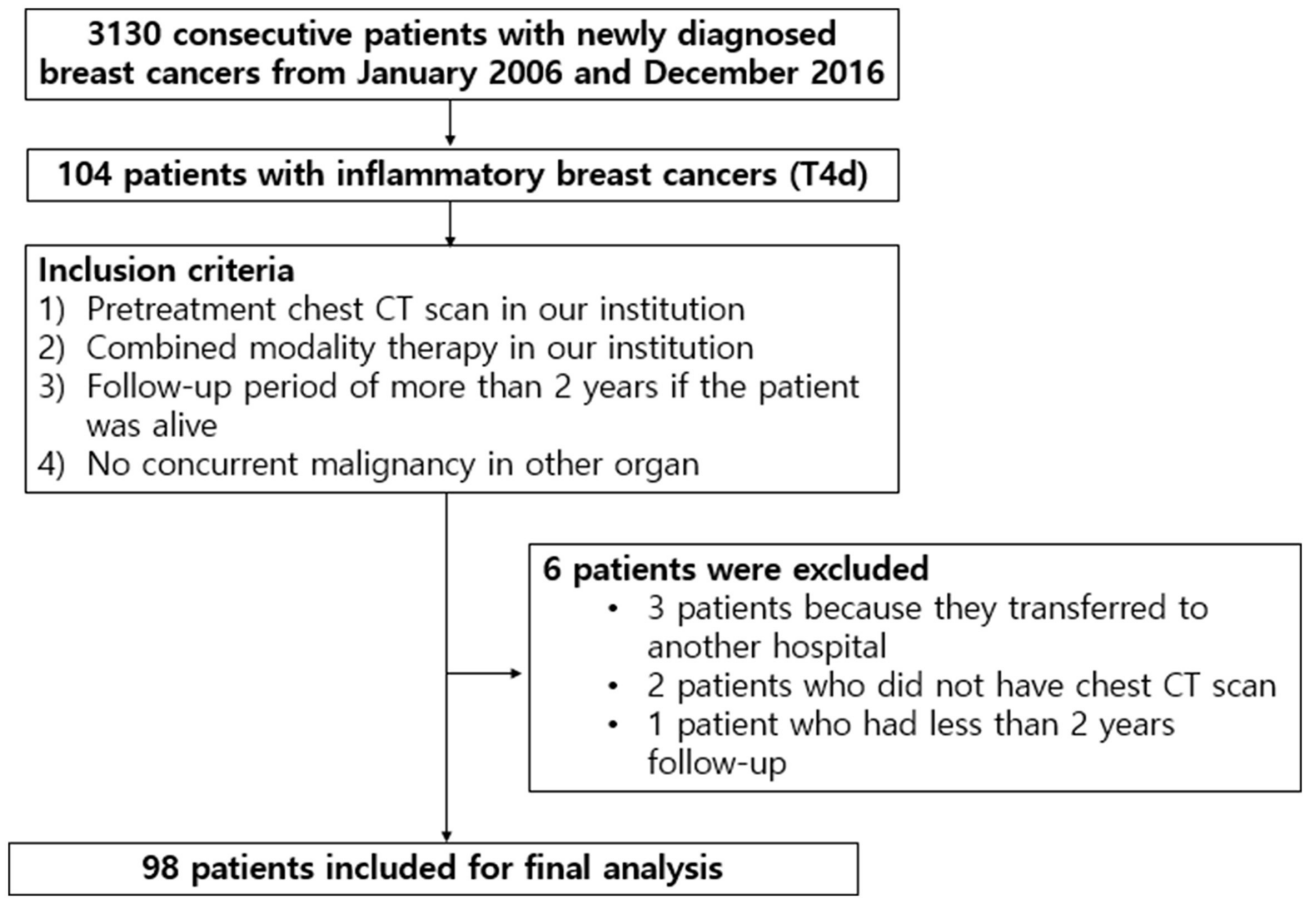
A flow diagram of study population.

This retrospective study was approved by our institutional review board (IRB Number: 2020AS0113), and the need for written informed patient consent was waived. After a review of medical records between January 2006 and December 2016, we identified 3,130 consecutive patients with breast cancer who were first diagnosed with tissue biopsies in our institution. Among those, 104 patients (3.3%) had IBC in AJCC breast cancer staging (Giuliano et al., 2018). The inclusion criteria were as follows: (i) pretreatment contrast-enhanced chest CT scan in our institution; (ii) combined modality therapy in our institution, consisting of neoadjuvant chemotherapy, radiotherapy with or without surgery, and/or adjuvant chemotherapy; (iii) a follow-up period of more than 2 years if the patient was alive; and (iv) no concurrent malignancy in other organs. Of the 104 patients, six were excluded: three patients because they were transferred to another hospital, two patients who did not have chest CT images, and one patient had <2 years of follow-up. Final study population, consisting of 98 patients, was presented in Figure 1. The interval between pathological diagnosis of breast cancer and chest CT scans ranged from 3 to 30 days (median: 7 days).

### CT Acquisition

Various CT scanners were used, including Brilliance 64 or Ingenuity 128 or Icon Spectral CT (Philips Medical Systems, Amsterdam, Netherlands), or Somatom Volume Zoom or Somatom Definition Flash (Siemens, Erlangen, Germany). Our chest CT was performed, using tube voltage of 80–120 kVp and tube current ranging from 30 to 300 mAs. The acquired CT images were reconstructed into a lung and a mediastinal window. The reconstruction parameters comprised of a 3-mm-slice thickness and no reconstruction interval. For the post-contrast scan, intravenous contrast medium (120–150 ml of 300–370 mg/ml non-ionic contrast) was injected at a rate of 2–3 ml/s, followed by a 40-m saline bolus. Scanning was commenced 90 s after contrast medium injection.

### CT Texture Analysis

**Figure 2:**
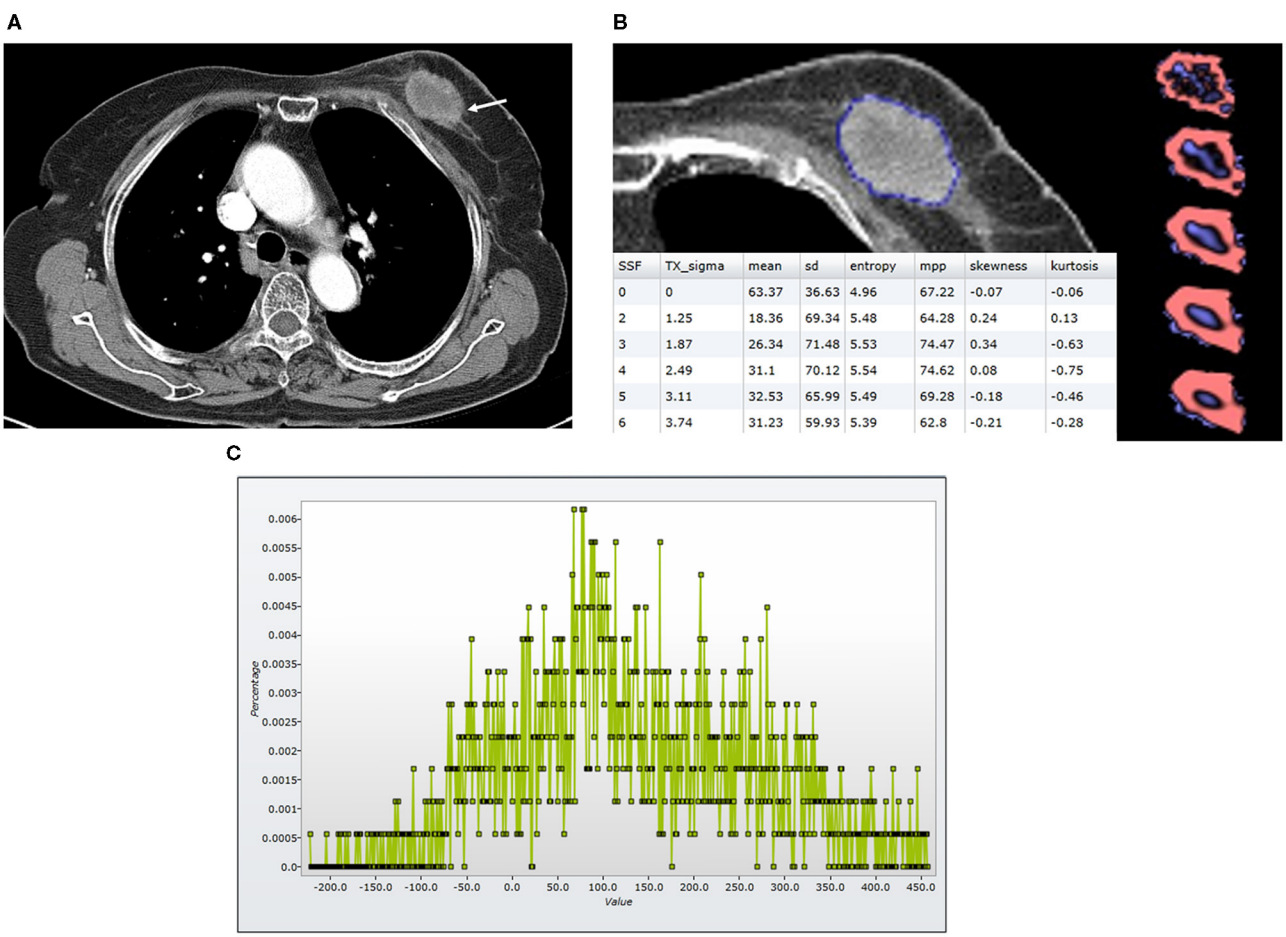
Findings in a 74-year-old woman who died 23 months after the diagnosis of luminal-like breast cancer in the left breast. At the time of diagnosis, she did not have a distant metastasis. **(A)** An axial post-contrast CT image shows an irregular enhancing mass (arrow) with a central area of low attenuation, suggesting necrosis. **(B)** A texture analysis image shows a region of interest (blue line), texture maps, and texture parameters on the CT image. **(C)** A CT texture histogram is obtained; from which, the different statistical-based metrics are extracted. Mean, MPP, entropy, and SD were lower for the patient with poor a prognosis. MPP, mean of positive pixels; SD, standard deviation.

**Figure 3:**
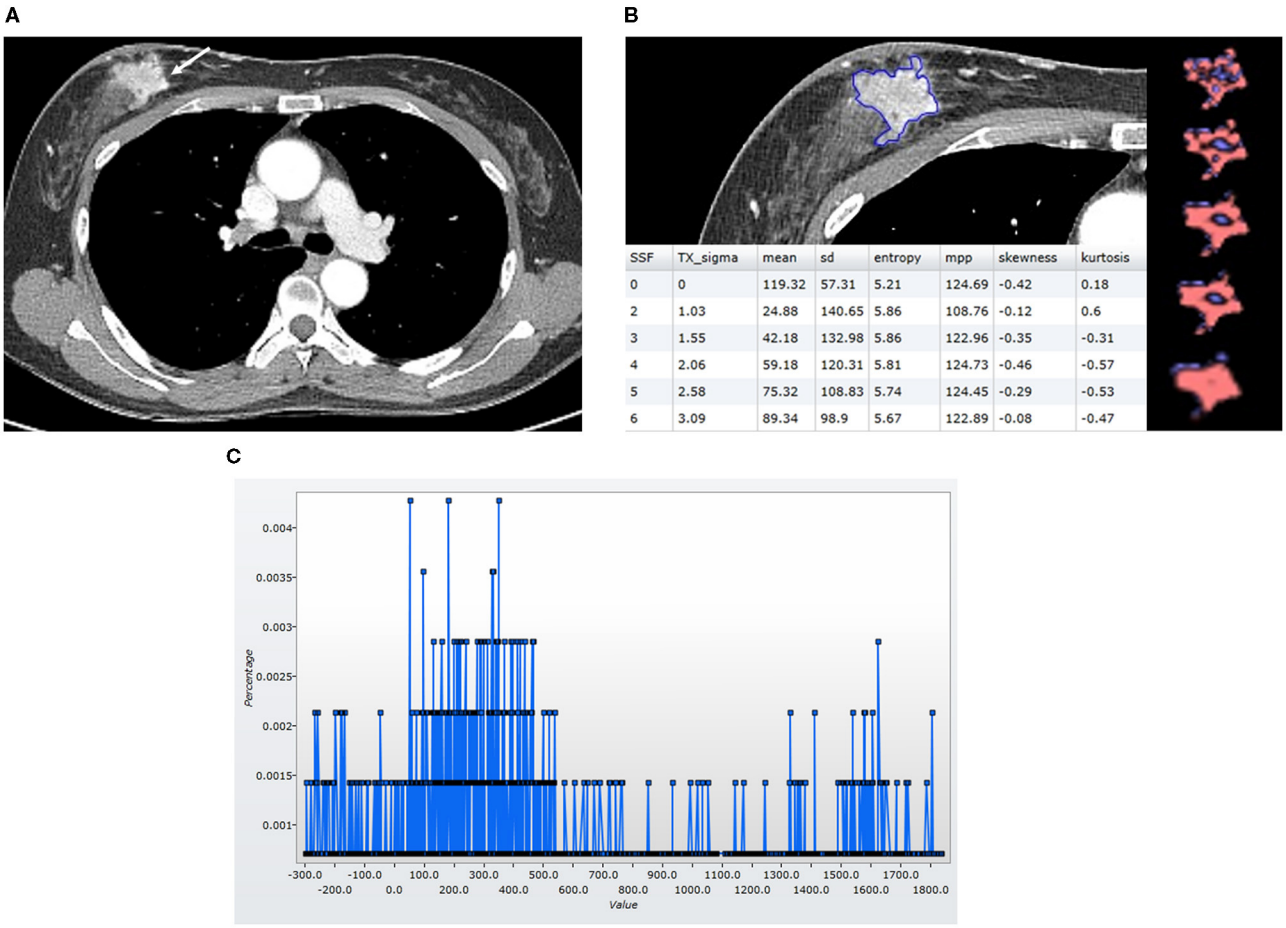
Findings in a 49-year-old woman who was alive 65 months after the diagnosis of luminal-like breast cancer in the right breast. At the time of the diagnosis, she did not have a distant metastasis. **(A)** An axial post-contrast CT image shows an irregular mass (arrow) with strong enhancement. **(B)** A texture analysis image shows a region of interest (blue line), texture maps, and texture parameters on the CT image. **(C)** A CT texture histogram is obtained; from which, the different statistical-based metrics are extracted. Mean, MPP, entropy, and SD were higher for the patient with a good prognosis. MPP, mean of positive pixels; SD, standard deviation.

Histogram-based texture analysis was done on the axial post-contrast DICOM images with a mediastinal window setting, using commercially available research software (TexRAD software—Feedback Medical Ltd., Cambridge, UK). Two radiologists (S.E.S. and B.K.S., 10 and 22 years of experience in breast imaging), who were blinded to the clinicopathologic findings and survival outcomes, reviewed the CT images and drew a region of interest (ROI) two times on the CT image surrounding the entire enhancing tumor margin at the largest cross-sectional area of the tumor (Figures 2, 3). For diffuse infiltrative tumors, the largest enhancing lesion was selected and measured. The initial step of texture analysis constitutes an image filtration-histogram step where the Laplacian of Gaussian band-pass filter was used to selectively extract textural features of different sizes and intensity variation. For tuning the filter parameter, spatial-scale image filtration (SSF), ranging from 2.0 to 6.0, was used. Each SSF corresponded to the same number of millimeters of pixel scales in radius; fine (SSF 2.0: 2 mm), medium (SSF 3.0–5.0: 3–5 mm), and coarse (SSF 6.0: 6 mm). After an image filtration-histogram step, a texture quantification step, which quantified heterogeneity within ROI with and without image filtration, using SSF, was performed for calculating six textural features (mean attenuation, standard deviation [SD], mean of positive pixels [MPP], entropy, kurtosis, and skewness) of the pixel distribution histogram. Mean attenuation is the average attenuation value (Hounsfield units, HU). SD is a measure of the dispersion from the average value of the histogram. MPP represents the average attenuation value of pixels >0, reflecting the average brightness of only positive pixel values. Entropy is a measure of irregularity or complexity. Kurtosis indicates the pointiness/sharpness of the histogram pixel distribution. Skewness reflects the asymmetry of the histogram pixel distribution. The values initially measured by one reader were used for statistical analysis. CT-based texture analysis has undergone the qualification and validation processes as evidenced from a number of publications in literature in an oncological setting (Van der Auwera et al., 2004; Ganeshan et al., 2012; Ng et al., 2013).

### Clinicopathologic Data

We evaluated AJCC breast cancer staging and pathological biomarkers, using the medical records database. Cancer staging was assessed as tumor, lymph node (N), and the metastasis (M) stage from physical examination and imaging studies, such as breast MRI, chest CT scan, bone scan, and positron emission tomography combined with CT scan. Estrogen receptor (ER), progesterone receptor (PR), human epidermal growth factor receptor 2 (HER2) status were evaluated from tissue biopsies or surgical specimens. ER- and PR-positivities were determined, using a cutoff value of >1% positively stained nuclei. Silver-enhanced *in situ* hybridization (SISH) was performed to detect the presence of HER2 amplification (gene copy number >6 or HER2/chromosome 17 ratio >2.2) if tumors had HER2 scores of 2+. On the basis of the results of ER, PR, and HER2 analysis, the tumors were categorized into three molecular subtypes: luminal-like (ER/PR-positive and HER2-negative), HER2-like (ER/PR-negative and HER2-positive), and basal-like (ER/PR/HER2-negative) (Giuliano et al., 2018). Treatment information, such as neoadjuvant chemotherapy, adjuvant chemotherapy, and adjuvant radiation therapy, was also recorded.

### Statistical Analysis

The primary end point was OS. Patients were followed up until death from disease progression or until March 2020 if they were alive. OS was defined as the time span from the date of diagnosis of IBC to the date of death or the last clinical follow-up.

All data were checked, using Levene's test for equality of variance. The textural features were compared between survivors and non-survivors, using non-parametric Mann–Whitney *U*-test. For survival analysis, significant textural features were dichotomized based on the optimal cutoff values identified by receiver operating characteristic curve analysis. The patients with values for textural features below the cutoff values were categorized into one group, while the remaining patients were categorized into another group. Kaplan–Meier curves were generated to compare survival between the two groups. Differences between the curves were evaluated by a log-rank test.

A univariate Cox proportional hazards model was used to analyze the effects on OS of clinicopathologic variables (age, *N* stage, *M* stage, ER, PR, HER2, molecular subtype, neoadjuvant chemotherapy, adjuvant chemotherapy, and adjuvant radiation therapy) and textural parameters. Collinearity between textural features was analyzed, using the Spearman rank correlation test, and a multivariate Cox proportional hazards model assessed the independence of each significant univariate texture predictor from the most significant clinical predictor of OS. Interobserver reliability between two readers and intraobserver reliability were assessed, using the interclass correlation coefficient (ICC). An *r* value of 1. was considered a perfect agreement; 0.81–0.99, almost perfect agreement; 0.61–0.80, substantial agreement; 0.41–0.60, moderate agreement; 0.21–0.40, fair agreement; and ≤0.20, slight agreement. Statistical analyses were performed, using SPSS version 20.0 (IBM Corp., Armonk, NY, USA), with a *P*-value < 0.05 considered to be significant.

## Results

### Patient Characteristics

Ninety-eight patients (mean age, 54 ± 12 years; range, 25–87 years) were evaluated. The most common clinical N stage was N3 (35.7%, 35 of 98), followed by N1 (34.7%, 34 of 98), N2 (25.5%, 25 of 98), and N0 (4.1%, 4 of 98). At the time of diagnosis, 38 patients (38.7%, 38 of 98) had M1 stage. Molecular subtypes were as follows: luminal-like (32.6%, 32 of 98), HER2-like (39.8%, 39 of 98), and basal-like (27.6%, 27 of 98). Of the 98 patients, 88 (89.7%) underwent mastectomy, but the remaining 10 (10.3%) did not undergo surgery because of their poor general condition. During a mean follow-up period of 47.9 months (range, 3–157 months), 41 of 98 patients (41.8%) died with a median OS of 20.0 months (range, 3–62 months). Among the clinicopathologic variables, M1 was the only significant predictor of OS (*P* < 0.001). These data are summarized in Table 1.

**Table 1:** Univariate cox proportional hazard analysis of clinicopathologic variables associated with worse overall survival.

		**Overall survival**	
**Variable**	**No. of patients**	**Hazard ratio**	***P*-value**
**Age**			
≥45 years	81	Reference	
<45 years	17	1.40 (0.64, 3.12)	0.392
***N*** **stage**			
0–1	38	Reference	
2–3	60	1.67 (0.86, 3.24)	0.125
***M*** **stage**			
0	60	Reference	
1	38	4.05 (2.14, 7.65)	<0.001
**ER**			
Positive	44	Reference	
Negative	54	1.12 (0.56, 1.96)	0.891
**PR**			
Positive	37	Reference	
Negative	61	1.06 (0.56, 2.00)	0.867
**HER2**			
Positive	39	Reference	
Negative	59	1.54 (0.80, 3.03)	0.196
**Molecular subtype**			
Luminal-like		Reference	
HER2-like		0.63 (0.30, 1.30)	0.215
Basal-like		0.94 (0.44, 1.99)	0.876
**Neoadjuvant chemotherapy**			
Not done	24	Reference	
Done	74	1.68 (0.74, 3.80)	0.211
**Adjuvant chemotherapy**			
Not done	5	Reference	
Done	93	0.91 (0.22, 3.78)	0.899
**Adjuvant radiation therapy**			
Not done	5	Reference	
Done	93	1.02 (0.51, 2.04)	0.273

*Data in parentheses are 95% confidence intervals. ER, estrogen receptor; PR, progesterone receptor; HER2, human epidermal growth factor receptor 2*.

### CT Texture Analysis and Survival Outcomes

**Figure 4:**
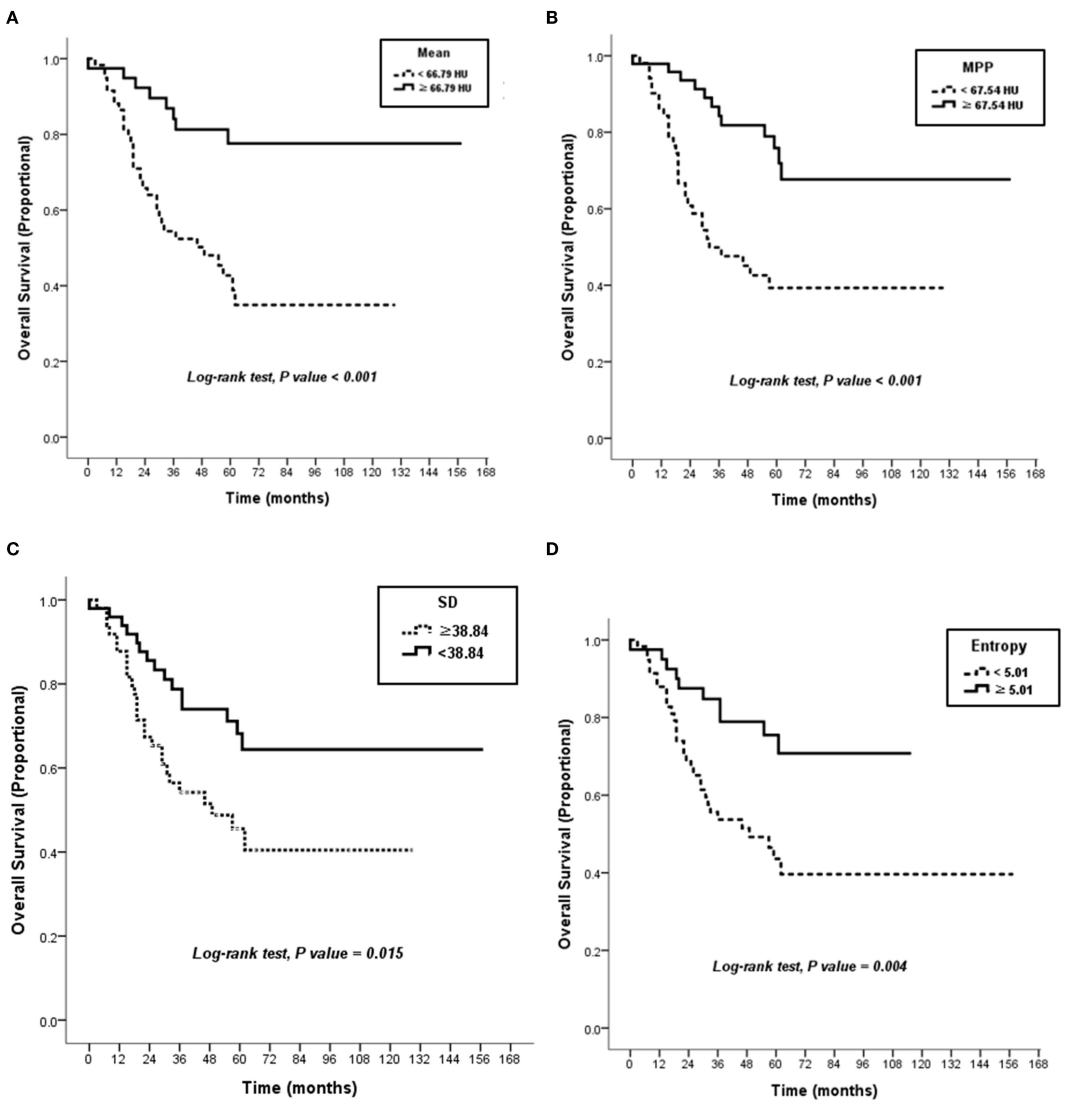
Kaplan–Meier curves. There were significant differences in overall survival according to attenuation of 66.79 HU in mean on post-contrast the CT image (*P* < 0.001) **(A)**, attenuation of 67.54 HU in MPP on the CT image (*P* < 0.001) **(B)**, SD of 38.84 on the CT image (*P* = 0.015) **(C)**, and 5.01 in entropy on the CT image (*P* = 0.004) **(D)**. HU, Hounsfield units; MPP, mean of positive pixels; SD, standard deviation.

The CT textural features were compared between survivors (*n* = 57) and non-survivors (*n* = 41). When no image filtration (SSF0) was used, non-survivors showed lower mean attenuation, SD, MPP, and entropy on CT images (all *P* < 0.05) (Table 2). However, textural features obtained using an SSF of 2–6 showed no differences between survivors and non-survivors (Supplementary Table 1). Receiver operating characteristic curve analyses determined that the optimal cutoff values were 66.79 HU (area under the curve [AUC], 0.67; *P* = 0.004) for mean attenuation, 38.84 (AUC, 0.63; *P* = 0.024) for SD, 67.54 (AUC, 0.69; *P* = 0.001) for MPP, and 5.01 (AUC, 0.62; *P* = 0.042) for entropy on CT images (Supplementary Figure 1). Using these cutoffs, textural features were categorized into two groups. Kaplan–Meier curves were generated to compare OS between the two groups, and the log-rank test revealed that the textural features were significantly associated with OS outcomes (Figure 4). The Kaplan–Meier curve of *M* stage was shown in Supplementary Figure 2.

**Table 2:** Comparison of CT texture features between survivors and non-survivors.

**Texture feature**	**All patients (*n* = 98)**	**Survivors (*n* = 57)**	**Non-survivors (*n* = 41)**	***P*-value**
Mean	60.92 ± 22.24	66.44 ± 23.14	53.25 ± 18.60	0.003
SD	40.91 ± 11.97	43.55 ± 13.48	37.24 ± 8.35	0.005
MPP	68.61 ± 19.65	74.33 ± 20.77	60.66 ± 14.87	<0.001
Entropy	4.96 ± 0.29	5.01 ± 0.32	4.89 ± 0.23	0.031
Skewness	−0.51 ± 0.81	−0.41 ± 0.70	−0.65 ± 0.93	0.157
Kurtosis	2.63 ± 6.95	1.88 ± 6.14	3.66 ± 7.99	0.232

*Data are mean ± standard deviation. SSF, spatial scale filters; SD, standard deviation; MPP, mean of positive pixels*.

### Survival Analysis Using Cox Proportional Hazard Models

Among the textural features, lower mean attenuation (hazard ratio [HR], 3.87; *P* = 0.001), lower SD (HR, 2.18; *P* = 0.016), lower MPP (HR, 3.31; *P* = 0.001), and lower entropy (HR, 2.78; *P* = 0.005) on CT images were associated with worse OS in univariate analysis (Table 3). Collinearity was observed between all the textural features (all, *P* < 0.05) (Supplementary Table 2). Because of this collinearity, each dichotomized CT textural feature was analyzed with M1 stage, using a multivariate Cox proportional hazards model. In multivariate analysis, lower mean attenuation (HR, 3.26; *P* = 0.003), lower MPP (HR, 3.03; *P* = 0.002), and lower entropy (HR, 2.70; *P* = 0.009) on post-contrast CT images were significant factors in predicting worse OS, independent of M1 stage (Table 4).

**Table 3:** Univariate Cox proportional hazard analysis of CT texture features associated with worse overall survival.

		**Overall survival**	
**Texture feature**	**No. of patients**	**Hazard ratio**	***P*-value**
**Mean**			
≥66.79 HU	39	Reference	
<66.79 HU	59	3.87 (1.78, 8.43)	0.001
**SD**			
≥38.84	49	Reference	
<38.84	49	2.18 (1.15, 4.13)	0.016
**MPP**			
≥67.54	47	Reference	
<67.54	51	3.31 (1.67, 6.52)	0.001
**Entropy**			
≥5.01	40	Reference	
<5.01	58	2.78 (1.36, 5.69)	0.005

*Data in parentheses are 95% confidence intervals. SSF, spatial scale filters; HU, Hounsfield unit; SD, standard deviation; MPP, mean of positive pixels*.

**Table 4:** Multivariate Cox proportional hazard analysis of clinicopathologic variables and CT texture features without image filtration associated with worse overall survival.

	**Overall survival**	
**Variable**	**Hazard ratio**	***P*-value**
***M*** **stage**		
0	Reference	
1	3.53 (1.85, 6.74)	<0.001
**Mean**		
≥66.79 HU	Reference	
<66.79 HU	3.26 (1.48, 7.16)	0.003
***M*** **stage**		
0	Reference	
1	3.68 (1.93, 7.01)	<0.001
**SD**		
≥38.84	Reference	
<38.84	1.77 (0.92, 3.38)	0.083
***M*** **stage**		
0	Reference	
1	3.81 (1.99, 7.28)	<0.001
**MPP**		
≥67.54	Reference	
<67.54	3.03 (1.52, 6.05)	0.002
***M*** **stage**		
0	Reference	
1	3.79 (2.00, 7.18)	<0.001
**Entropy**		
≥5.01	Reference	
<5.01	2.70 (1.28, 5.69)	0.009

*Data in parentheses are 95% confidence intervals. HU, Hounsfield unit; SD, standard deviation; MPP, mean of positive pixels*.

### Interobserver and Intraobserver Agreement

For interobserver agreement, the ICC between two readers was calculated. The ICC was 0.77 for mean, 0.81 for SD, 0.82 for entropy, 0.80 for MPP, 0.28 for skewness, and 0.16 for kurtosis. For intraobserver agreement, the ICC for reader 1 was 0.98 for mean, 0.93 for SD, 0.98 for entropy, 0.99 for MPP, 0.32 for skewness, and 0.18 for kurtosis, and that for reader 2 was 0.95 for mean, 0.90 for SD, 0.91 for entropy, 0.95 for MPP, 0.30 for skewness, and 0.19 for kurtosis.

## Discussion

Our results suggest that CT-based texture analysis can assess tumor heterogeneity and provide prognostic information in patients with IBC. Histogram-based statistical parameters, such as lower mean attenuation, lower MPP, and lower entropy on post-contrast CT images, were significant predictors of worse OS in the patients with IBC, independent of M1 stage at the time of diagnosis.

Our study had strengths. First, our exploratory study is the first to report the value of simple CT-based histogram statistics to act as a prognostic indicator for IBC, which can potentially be readily incorporated in routine practice. Since we used commercial software for image analysis, the results can be applied to clinical practice and multicenter studies. Our results suggest that CT-based texture analysis can be used as an adjunct in risk stratification for patients with IBC, which could help in making optimal treatment decisions and improve overall clinical outcomes. An interesting finding of our study is that when no image filtration was used, the texture features were different between survivors and non-survivors, while, when image filtration was used, there was no significant difference. Our results implicate that simple histogram statistics that do not require a dedicated post-processing platform can be used as prognostic markers in patients with breast cancer. This will increase the utility and applicability of CT-based texture analysis for patients with breast cancer. Second, our study provided further insight into the interpretation of texture results for the design of future CT studies in breast malignancies and non-measurable tumors according to the RECIST guideline (Eisenhauer et al., 2009). At RECIST, tumor lesions are categorized as measurable or non-measurable. Non-measurable lesions include IBC, leptomeningeal disease, ascites, pleural or pericardial effusion, lymphangitic involvement of the lung, and abdominal masses identified by a physical examination that cannot be measured by imaging techniques. Future studies could look at more intrinsic and complex texture analysis and machine-learning algorithms.

IBC is highly angiogenic and lymphangiogenic by gene expression quantification and immunohistochemistry, compared with other breast cancer types (McCarthy et al., 2002; Shan et al., 2013). Therefore, it has the potential to metastasize through both the hematogenous and lymphatic routes, and shows extensive spread to the breast subcutaneous lymphatics. Our results may be related to these pathologic properties of IBC. Lower mean CT attenuation in IBC is indicative of extensive necrosis, resulting from rapid tumor growth and edematous swelling from lymphatic emboli. Shan et al. (2013) evaluated the correlation between the degree of necrosis on CT images and the expression of hypoxic and angiogenesis biomarkers in breast cancers, and demonstrated that lower CT values were significantly positively correlated with microvessel density and hypoxic biomarkers. Rapid expansion of a tumor mass requires neovascularization to provide adequate oxygen and metabolites to the proliferating tumor cells; the absence of which results in hypoxia. Perrone et al. (2008) reported that the mean CT attenuation of 27 non-inflammatory breast cancers was 125 HU on post-contrast images. In our study, the mean attenuation of IBC was 60.92 HU on post-contrast images, lower than the values for non-inflammatory breast cancers (Perrone et al., 2008), which might have resulted from tumor necrosis and hypoxia. A CT texture study of metastatic renal cell carcinomas (Matoori et al., 2017) reported that lower mean attenuation predicted poor OS, and that pretreatment mean attenuation was a predictive marker of treatment response. Similarly, a lower mean CT attenuation of the tumor might be useful as an imaging biomarker for predicting worse OS in patients with IBC.

In our study, lower MPP predicted worse OS in patients with IBC. This finding is consistent with previous reports, demonstrating that lower MPP was associated with poor survival outcomes in patients with non-small cell lung cancer and hepatic metastatic colorectal cancer (Lubner et al., 2015; Hayano et al., 2016). Several studies have reported that MPP is associated with tumor angiogenesis, grade, and necrosis (Ganeshan et al., 2013; Lubner et al., 2015; Tian et al., 2015). In non-small cell lung cancer, MPP showed a significant inverse association with CD34 expression, which is an immunohistochemial marker of angiogenesis (Ganeshan et al., 2013). In hepatic metastatic colorectal cancers, lower MPP, SD, entropy on post-constrast images were associated with higher tumor grade (Lubner et al., 2015). In addition, because MPP was strongly associated with tumor necrosis, it was suggested to be the best predictor for chemotherapy response in soft-tissue sarcomas (Tian et al., 2015). In our study, lower MPP may be related to more extensive lymphovascular proliferation and necrosis in patients with IBC with poor OS. Further studies will be needed to confirm the association between MPP and pathological characteristics of IBC.

Entropy indicates irregularity and a marker of tumor heterogeneity. In our study, lower entropy on post-contrast CT images was associated with poor OS in patients with IBC. Previous CT texture studies of colon cancer and hepatic metastatic colorectal cancers also showed a relationship between lower entropy and poorer survival (Ng et al., 2013; Lubner et al., 2015). Kim et al. (2017) investigated MRI textural features in breast cancer and demonstrated that lower entropy on post-contrast T1-weighted images and higher entropy on T2-weighted images were associated with worse recurrence-free survival. They inferred that tumors with greater heterogeneity (higher entropy on T2-weighted images) could show more homogeneous enhancement on post-contrast T1-weighted images because of increased permeability; greater permeability that leads to lower contrast resolution between parenchyma and adjacent blood vessels may represent as lower heterogeneity on post-contrast images. Therefore, based on the results of previous MRI texture studies and our CT study, we suppose that lower entropy on post-contrast CT or MRI may be associated with worse OS in breast cancer patients.

Metastasis is common at the time of diagnosis of IBC because the increased lymphovascular proliferation and vascular permeability of IBC promote a distant metastasis. A large-cohort study of IBC (Wang et al., 2020) revealed that 224 of 635 patients (35.3%) had a distant metastasis, which was very similar to the 38.7% seen in our study. In our study, a metastasis had a significant impact on OS; however, known prognotic factors, such as lymph node involvement, hormone receptor negativity or age (Chevallier et al., 1987; Fields et al., 1989; Palangie et al., 1994), were not associated with worse OS.

Our study had several limitations. Firstly, the mean follow-up period of the study population was short at 47.9 months; that of non-survivors was 25.8 months, and that of survivors was 63.0 months. A longer follow-up period will be required to acquire a robust estimate of the impact of texture features on the survival outcome. Secondly, we obtained texture features on a single section of the largest cross-sectional diameter of the tumor, rather than on the multi-slice or volume analysis. However, according to previous studies, the texture results from two-dimensional and three-dimensional analysis are similar, and the intraobserver reproducibility of single-section measurements is quite high (Lubner et al., 2015; Smith et al., 2015). Based on our results, ICCs for mean, SD, entropy, and MPP, which were significant predictors for OS, showed almost perfect to perfect agreement. On the other hand, ICCs for skewness and kurtosis showed slight to a fair agreement. Thirdly, our study was retrospective in nature and results were acquired from a single institution, using a single software program on a small sample size. Finally, we included the patients with IBC with the M1 stage in our study population despite the M1 stage is a well-known factor predicting a worse prognosis. We collected the patients with IBC during 10 years, but the number of study population was small, and 38.7% of the patients with IBC (38 of 98) had M1 stage at the time of diagnosis. Nevertheless, our study proved that texture features were significant factors independent from the M1 stage for predicting worse OS in patients with IBC.

In conclusion, our results from an exploratory study highlight the potential of CT-based texture analysis to act as a non-invasive imaging biomarker, predicting the prognosis of patients with IBC. Identifying new risk factors from chest CT scans may allow clinicians to optimize surveillance and treatment strategies based on individual risk of the patients with IBC.

## Data Availability Statement

The original contributions presented in the study are included in the article/Supplementary Material, further inquiries can be directed to the corresponding author.

## Ethics Statement

The studies involving human participants were reviewed and approved by Korea University Ansan Hospital IRB committee. Written informed consent for participation was not required for this study in accordance with the national legislation and the institutional requirements.

## Author Contributions

BKS was responsible for research design, research process management, and financial support. SES performed image analysis. JC and BG performed the statistical analysis. OHW and KRC interpreted the results. SES and BKS drafted the manuscript. OHW and ESK made substantial contributions to the experimental design, data interpretation, and manuscript. All authors read and approved the final draft of the manuscript and are accountable for all aspects of the work.

## Conflict of Interest

BG is the Co-Founder/Co-Inventor of TexRAD texture analysis software used in this study and a shareholder (not an employee) of Feedback Plc., a UK based company which owns, develops and markets the TexRAD texture analysis software. The remaining authors declare that the research was conducted in the absence of any commercial or financial relationships that could be construed as a potential conflict of interest.

## Supplementary Material

Supplementary Figure 1 Receiver operating characteristic (ROC) curve analyses for the optimal cutoff values. An ROC curve (an area under the curve [AUC], 0.67; *P* = 0.004) for mean attenuation **(A)**, an ROC curve (AUC, 0.63; *P* = 0.024) for SD **(B)**, an ROC curve (AUC, 0.69; *P* = 0.001) for MPP **(C)**, and an ROC curve (AUC, 0.62; *P* = 0.042) for entropy **(D)**.

Supplementary Figure 2 Kaplan–Meier curves of the *M* stage. There were significant differences in overall survival according to the metastasis (M1) stage at the time of the diagnosis.

Supplementary Table 1 CT texture features at spatial scale filters of 2–6.

Supplementary Table 2 Spearman rank correlation coefficients for CT texture features.
