# Supplementary figures and images for: Prediction of Inflammatory Breast Cancer Survival Outcomes Using Computed Tomography-Based Texture Analysis
Source: Front Bioeng Biotechnol. 2021 Jul 20;9:695305. doi: 10.3389/fbioe.2021.695305 (PMC8329959; doi:10.3389/fbioe.2021.695305)

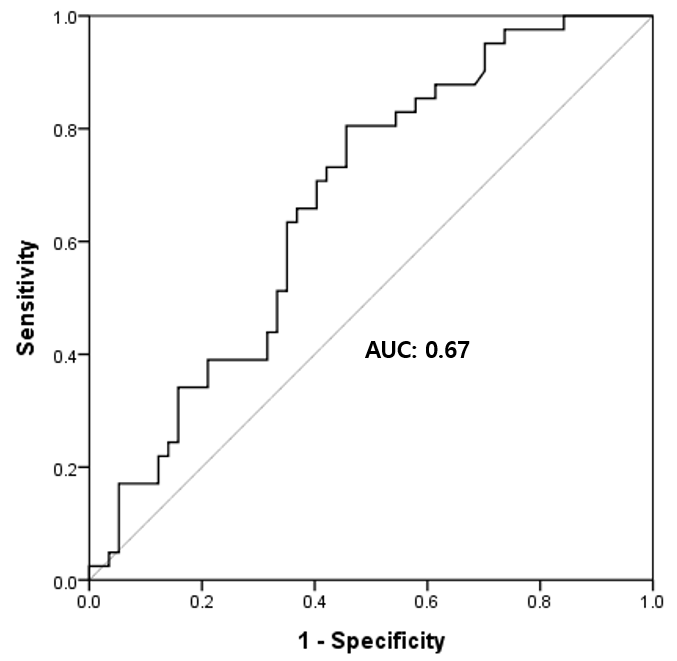

Supplement: Supplementary Figure 1 — Receiver operating characteristic (ROC) curve analyses for the optimal cutoff values. An ROC curve (an area under the curve [AUC], 0.67; P = 0.004) for mean attenuation (A), an ROC curve (AUC, 0.63; P = 0.024) for SD (B), an ROC curve (AUC, 0.69; P = 0.001) for MPP (C), and an ROC curve (AUC, 0.62; P = 0.042) for entropy (D). [file Image_1.TIF]

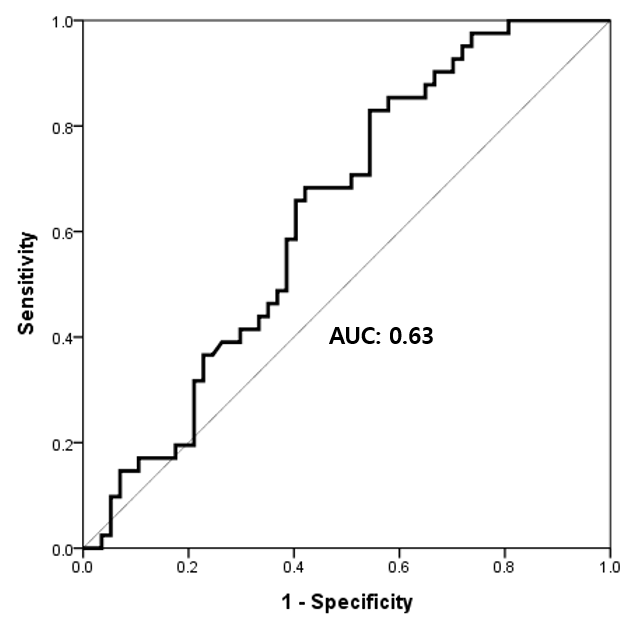

Supplement: Supplementary Figure 2 — Kaplan–Meier curves of the M stage. There were significant differences in overall survival according to the metastasis (M1) stage at the time of the diagnosis. [file Image_2.TIF]

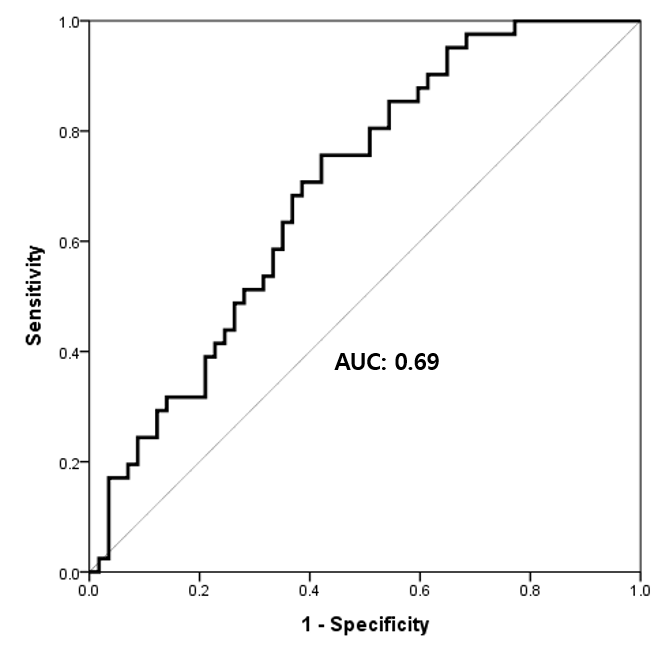

Supplement: Supplementary file 4 [file Image_3.TIF]

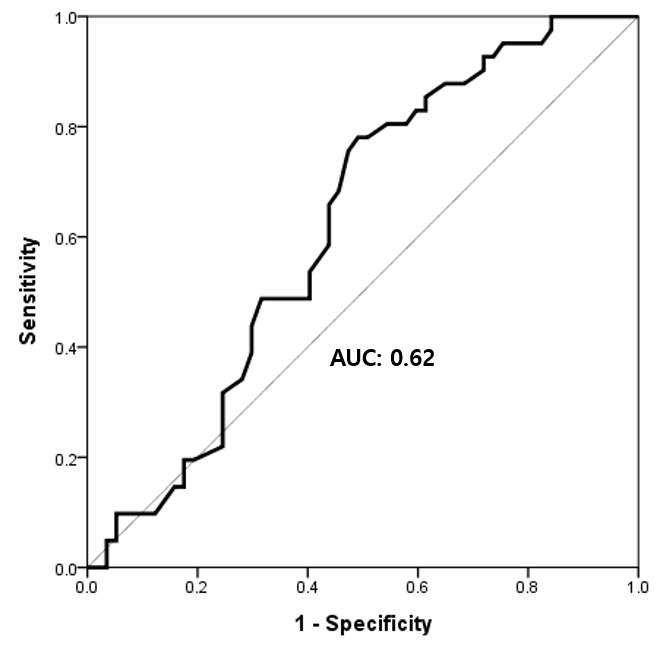

Supplement: Supplementary file 5 [file Image_4.TIF]

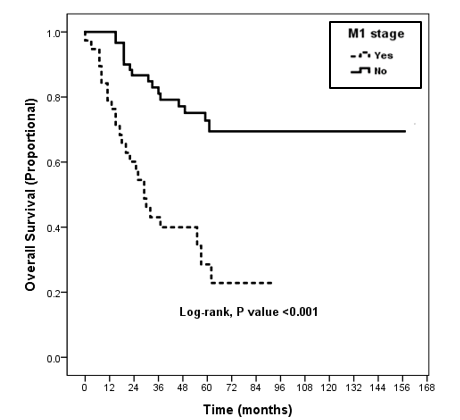

Supplement: Supplementary file 6 [file Image_5.TIF]
