# Supplementary material for: Prediction of Inflammatory Breast Cancer Survival Outcomes Using Computed Tomography-Based Texture Analysis
Source: Front Bioeng Biotechnol. 2021 Jul 20;9:695305. doi: 10.3389/fbioe.2021.695305 (PMC8329959; doi:10.3389/fbioe.2021.695305)
Supplement: Supplementary Table 1 — CT texture features at spatial scale filters of 2–6. [file Table_1.DOCX]

**Supplementary Tables**

**Supplementary Table 1. CT Texture Features at Spatial Scale Filters of 2-6**

| **Texture features** | **Survivors**  **(n=57)** | **Non-survivors**  **(n=41)** | **P value** |
| --- | --- | --- | --- |
| SSF2 |  |  |  |
| Mean | 50.72±34.58 | 56.12±36.35 | 0.458 |
| SD | 123.37±68.59 | 140.36±80.76 | 0.264 |
| MPP | 105.37±47.80 | 114.50±52.47 | 0.373 |
| Entropy | 5.69±0.24 | 5.75±0.28 | 0.256 |
| Skewness | 1.44±1.63 | 1.60±1.80 | 0.653 |
| Kurtosis | 7.67±12.43 | 8.12±13.17 | 0.863 |
| SSF3 |  |  |  |
| Mean | 85.25±56.98 | 93.36±62.10 | 0.505 |
| SD | 155.98±96.53 | 177.26±117.76 | 0.329 |
| MPP | 140.56±75.33 | 154.07±86.52 | 0.413 |
| Entropy | 5.78±0.28 | 5.86±0.33 | 0.187 |
| Skewness | 1.59±1.69 | 1.48±1.64 | 0.753 |
| Kurtosis | 7.18±10.79 | 6.23±9.62 | 0.655 |
| SSF4 |  |  |  |
| Mean | 121.38±78.13 | 129.30±86.21 | 0.637 |
| SD | 189.36±120.13 | 207.50±144.12 | 0.499 |
| MPP | 178.39±97.61 | 190.98±115.82 | 0.562 |
| Entropy | 5.87±0.32 | 5.95±0.38 | 0.322 |
| Skewness | 1.38±1.49 | 1.49±1.57 | 0.734 |
| Kurtosis | 4.80±7.12 | 6.27±10.90 | 0.423 |
| SSF5 |  |  |  |
| Mean | 159.85±99.18 | 164.53±108.60 | 0.825 |
| SD | 220.45±141.07 | 234.45±162.10 | 0.650 |
| MPP | 217.78±117.13 | 225.13±139.43 | 0.778 |
| Entropy | 5.97±0.35 | 6.01±0.42 | 0.591 |
| Skewness | 1.25±1.26 | 1.50±1.63 | 0.384 |
| Kurtosis | 3.49±4.94 | 6.27±12.11 | 0.171 |
| SSF6 |  |  |  |
| Mean | 202.43±121.62 | 200.32±130.31 | 0.935 |
| SD | 248.61±158.51 | 259.89±175.13 | 0.740 |
| MPP | 258.58±136.15 | 259.20±158.56 | 0.983 |
| Entropy | 6.06±0.39 | 6.08±0.46 | 0.814 |
| Skewness | 1.12±1.09 | 1.34±1.49 | 0.435 |
| Kurtosis | 2.57±3.76 | 4.66±9.04 | 0.486 |

Data are mean$\pm$standard deviation. SSF, spatial scale filters; SD, standard deviation; MPP, mean of positive pixels.

**Supplementary Table 2. Spearman Rank Correlation Coefficients for CT Texture Features**

| **Texture features** | **Mean** | **SD** | **Entropy** | **MPP** |
| --- | --- | --- | --- | --- |
| Mean | … | 0.297 (0.003) | 0.287 (0.004) | 0.961 (<0.001) |
| SD | 0.297 (0.003) | … | 0.932 (<0.001) | 0.496 (<0.001) |
| Entropy | 0.287 (0.004) | 0.932 (<0.001) | … | 0.465 (<0.001) |
| MPP | 0.961 (<0.001) | 0.496 (<0.001) | 0.465 (<0.001) | … |

Data in parentheses are *P* values. SD, standard deviation; MPP, mean of positive pixels.
